# Insights from density functional theory calculations on heteroatom P-doped ZnIn_2_S_4_ bilayer nanosheets with atomic-level charge steering for photocatalytic water splitting

**DOI:** 10.1038/s41598-022-05740-8

**Published:** 2022-02-04

**Authors:** Wei-Kean Chong, Boon-Junn Ng, Chen-Chen Er, Lling-Lling Tan, Siang-Piao Chai

**Affiliations:** Multidisciplinary Platform of Advanced Engineering, Chemical Engineering Discipline, School of Engineering, Monash University, Jalan Lagoon Selatan , 47500 Bandar Sunway, Selangor Malaysia

**Keywords:** Chemical engineering, Energy infrastructure, Energy harvesting, Energy infrastructure, Renewable energy, Computational science

## Abstract

ZnIn_2_S_4_ (ZIS) is an efficient photocatalyst for solar hydrogen (H_2_) generation from water splitting owing to its suitable band gap, excellent photocatalytic behaviour and high stability. Nevertheless, modifications are still necessary to further enhance the photocatalytic performance of ZIS for practical applications. This has led to our interest in exploring phosphorus doping on ZIS for photocatalytic water splitting, which has not been studied till date. Herein, phosphorus-doped ZnIn_2_S_4_ (P-ZIS) was modelled via Density Functional Theory to investigate the effects of doping phosphorus on the structural and electronics properties of ZIS as well as its performance toward photocatalytic water splitting. This work revealed that the replacement of S3 atom by substitutional phosphorus gave rise to the most stable P-ZIS structure. In addition, P-ZIS was observed to experience a reduction in band gap energy, an upshift of valence band maximum (VBM), an increase in electron density near VBM and a reduction of H* adsorption–desorption barrier, all of which are essential for the enhancement of the hydrogen evolution reaction. In overall, detailed theoretical analysis carried out in this work could provide critical insights towards the development of P-ZIS-based photocatalysts for efficient H_2_ generation via solar water splitting.

## Introduction

Global energy demand is projected to increase substantially and is expected to hit a peak of 778 Etta Joule, or 18 million tonnes of oil equivalent by 2035^1–3^. Currently, more than 85% of global energy is produced by non-renewable fossil fuels such as oil, coal and natural gas^4,5^. As is widely known, the combustion of fossil fuels is the world’s largest contributor to air pollution and is driving global warming to dangerous levels. If no prompt action is taken now, future generations may grow up in a world made far more dangerous and unpredictable as a result of a changing climate and degraded environment. Therefore, the search for a greener and more sustainable energy source is inevitable. Solar energy serves as the most ideal energy source replacement owing to its abundance, wide distribution and non-polluting nature. To date, several technologies including photoelectric and photochemical systems have been employed to harvest green solar energy to produce renewable, pollutant-free and high energy content hydrogen (H_2_) fuel. These processes contribute to roughly 2% of the global energy and is projected to grow in years to come^6–9^^.^ More recently, incessant research attention has been directed to the utilization of photocatalysis, an artificial photosynthetic route, to produce H_2_ as an energy carrier. In this process, solar energy drives the splitting of water, leading to both hydrogen evolution reaction (HER) and oxygen evolution reaction (OER). When compared to photoelectric and photochemical systems, photocatalysis offers significant advantages due to its inexpensiveness and relatively simple design—The process only requires water and a suitable semiconductor photocatalyst to harvest solar energy for H_2_ generation^6,10–12^.

To date, a large number of research work has been carried out on different types of photocatalysts (e.g., metal oxides^13–15^, metal chalcogenides^16–18^ and g-C_3_N_4_^19,20^), along with various modification strategies to enhance their photocatalytic performances. Among all types of photocatalysts, two-dimensional (2D) bilayer zinc indium sulfide (ZnIn_2_S_4_ or ZIS) has attracted much research interests due to its high chemical stability, suitable band gap energy, facile synthesis, ease of modification and excellent photocatalytic activity. By adopting appropriate modification strategies, ZIS has the potential to provide superior photocatalytic efficiencies for practical HER applications in the energy sector^21^. ZIS, a ternary metal chalcogenide, is a layered structure semiconductor with a direct band gap that could exist in three different crystal polymorphs (cubic, trigonal, and hexagonal)^22^. Hexagonal ZIS has been reported to be the most thermodynamically stable structure with high photoactivity and is therefore given more focus in photocatalytic applications^23–27^. It exhibits a band gap energy (E_g_) of ~ 2.40 eV, with a conduction band minimum (CBM) of − 0.85 V (more negative than HER potential of 0 V) and a valence band maximum (VBM) of + 1.55 V (more positive than OER potential of + 1.23 V) against NHE potential at pH = 0^25^. Hence, ZIS is capable in utilizing a broad range of solar incident ray for the photoexcitation of electrons to drive photocatalytic water splitting. Despite the aforementioned merits, ZIS still suffers from several drawbacks such as high electron–hole recombination rate, sluggish diffusion of charge carriers to active sites and low exposure of active (110) facets for HER^21,27–30^. Such limitations inhibit the photo-redox reaction of water and deteriorate photocatalytic performances. Therefore, appropriate modifications must be done to improve intrinsic electronic properties, increase charge separation efficiency and promote the migration of charge carriers to active sites for initializing the redox reactions.

Elemental doping, either at substitutional or interstitial sites, involves the introduction of external heteroatoms into the crystal lattice of semiconductor. This addition could bring about new intermediate levels that reduce the band gap energy of the photocatalyst, thereby improving its photo-responsiveness towards solar spectrum^22,31^. Additionally, the presence of heteroatom dopants could alter the material’s electronic structure, which could in turn promoting the separation of charge carriers and their migration rates^32–34^. Phosphorus doping (P-doping) has been reported to show significant improvements on numerous semiconductors by accelerating the separation and transmission of photoinduced electron–hole pairs^35–42^. However, to the best of our knowledge, no prior research has been conducted to investigate the intrinsic nature and effects of P-doping on ZIS. Hence, in this work, Density Functional Theory (DFT) calculations were performed to investigate and elucidate the effects of P-doping on ZIS. The doping nature as well as the structural and electronic properties of P-ZIS, including its charge density distribution, band structure and density of state (DOS) were studied in detail. Systematic investigations were also carried out to examine the performance of P-ZIS towards water interaction, HER and OER.

## Computation detail and methodology

The theoretical structure was optimized using Vienna Ab initio Simulation Package (VASP) with projector augmented wave (PAW) method and created reciprocal space with plane-wave basis. DFT calculation was implemented in VASP, via generalization-gradient approximation (GGA) with exchange–correlation function of Perdew-Burke-Ernzerhof (PBE)^41^. Basic plane-wave settings were based on previously reported ZIS modelled structure in order to obtain consistent, comparable and convergence results. For instance, the energy cut-off was set at 500 eV. The position of all atoms and cell shapes were allowed to relax until an energy convergence of $1 \times 10^{ - 5}$ eV and force convergence of 0.01 eV/Å were obtained. During initialization calculation, 1-by-1 atomic bilayer with 2 Zn atoms, 4 In atoms and 8 S atoms was adapted. The Monkhorst–Pack k-point mesh was set at 3 × 3 × 1. Additional vacuum layer of approximately 15 Å was applied to eliminate possible interaction between periodic images^43^. Hybrid functional HSE06 was used to obtain the most stable structure during the relaxation calculation. For interstitial P-doping calculation, single P atom was added into possible interstitial sites of 1-by-1 atomic bilayer structure. Each intrinsic S atom was replaced by P atom during the substitutional P-doping calculation. In the calculation of the bandgap and DOS, high symmetry k-points of interest for ZIS structure were used including ${\Gamma }, \,{\text{M}},\, {\text{K}}$ and ${{\Gamma{A}}}$. All model lattice structures were visualized using Visualization for Electronic and Structural Analysis (VESTA).

For surface reaction study involving surface adsorption and active site interaction, single layer structure was utilized for investigation as recommended^28,41,44,45^. Herein, 1-by-1 atomic single layer ZIS (1 Zn atom, 2 In atoms and 4 S atoms) as well as P-ZIS (1 Zn atom, 2 In atoms, 3 S atoms and 1 P atom) structures were used for ease of convergence during surface reaction studies. 1.5 Å separation between active sites and adsorbed species were adopted for initialization calculation^42,46^. A number of active sites used in this study was chosen based on other reports^29^. During surface adsorption and interaction studies, additional Van der Waals (vdW) correction term utilizing D2 method of Grimme (DFT-D2) was accounted in computation of lattice relaxation to obtain accurate calculations in interatomic forces, potential energy and stress tensor^47^. Following that, free energy values calculated in DFT were utilized for analysis on both ZIS and P-ZIS structures towards HER, OER and water interactions, whereby adsorption energies and the corresponding Gibb’s free energies were calculated to conclude potential performance improvement (see Supplementary Information online for computation and calculation details).

## Results and discussion

### Pristine structure optimization and phosphorus doping simulation

**Figure 1:**
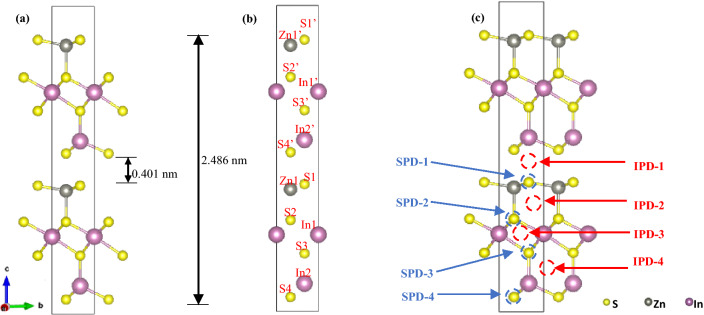
Crystal structure of 2D pristine bilayer ZnIn_2_S_4_ (**a**) with dimensions, and (**b**) excluding bonds for ease of atom visualization. Labelling of atoms are shown in (**b**) for the ease of discussion in subsequent parts. (**c**) Lattice representation of each doping site for interstitial phosphorus doping (IPD) and substitutional phosphorus doping (SPD).

To ensure that the correct structure was formed, several key parameters were cross-checked with literature values including basic lattice parameters $\left( {a = b = 3.85 {\AA}, \, c = 24.68 {\AA},\,\alpha = \beta = 90^\circ ,\,\gamma = 120^\circ } \right),$ bilayer thickness (~ 2.468 nm) and intermediate layer gap (~ 0.4 nm)^41,48^. Figures 1 and S1 show the simulated structures with different facets, which are consistent with those reported in the literature^41,48,49^. It should be noted that even though the simulated bilayer ZIS structure exhibited an identical arrangement of atoms at the top and bottom layers, the bond lengths of Zn1’-S1’ (top layer) and Zn1-S1 (bottom layer) differed due to potential interactions across the interlayer spacing. A similar argument can be made for the other bonds present. To confirm that the simulated ZIS structure was fully optimized, its powder diffraction pattern was obtained and compared with the X-ray Powder Diffraction (XRD) pattern from the literature (see Fig. S2)^49^**.** All characteristic $2\theta$ peaks from the simulated structure matched well with the literature values, with peaks at 21.5°, 27.6°, 30.4°, 39.7°, 47.1° and 52.4° corresponding to the indexed facets (006), (102), (104), (108), (110) and (120), respectively. Hence, it is clearly shown that the fully relaxed simulated structure is in line with the standard hexagonal ZIS structure.

DFT calculations were then conducted to analyse two different doping behaviours: (1) interstitial P-doping (IPD); and (2) substitutional P-doping (SPD), where the S atom was replaced due to its similar electronegativity and atomic radius of ~ 100 nm^41,42^. Figure 1c illustrates eight of the possible P-doping sites on ZIS. Table 1 summarizes the formation energy ($E_{f}$) of the eight possible P-doping sites (see Supplementary Information online for calculation details). It should be noted that the lowest $E_{f}$ calculated corresponded to the most stable and energetically favourable structure^41,50^.

**Table 1:** Summary of $E_{f}$ for each interstitial and substitutional doping site.

Doping Sites	${\varvec{E}}_{{\varvec{f}}}$(eV)	Doping Sites	${\varvec{E}}_{{\varvec{f}}}$(eV)
IPD-1	− 0.991	SPD-1	− 2.011
IPD-2	− 1.147	SPD-2	− 2.055
IPD-3	− 1.170	SPD-3	− 2.082
IPD-4	− 1.172	SPD-4	− 2.072

**Figure 2:**
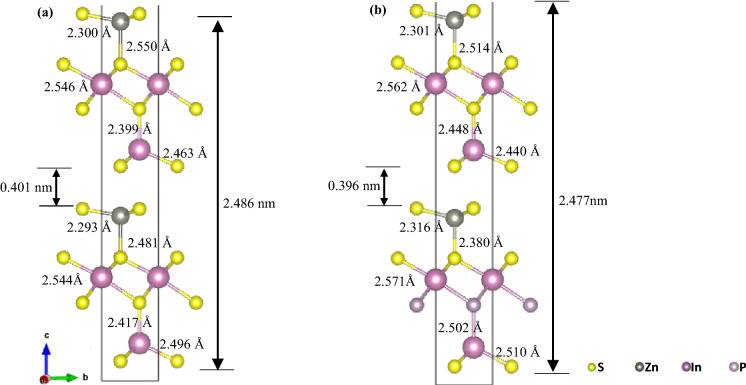
Simulated crystal structure for (**a**) pristine ZIS, and (**b**) P-ZIS (SPD-3).

**Figure 3:**
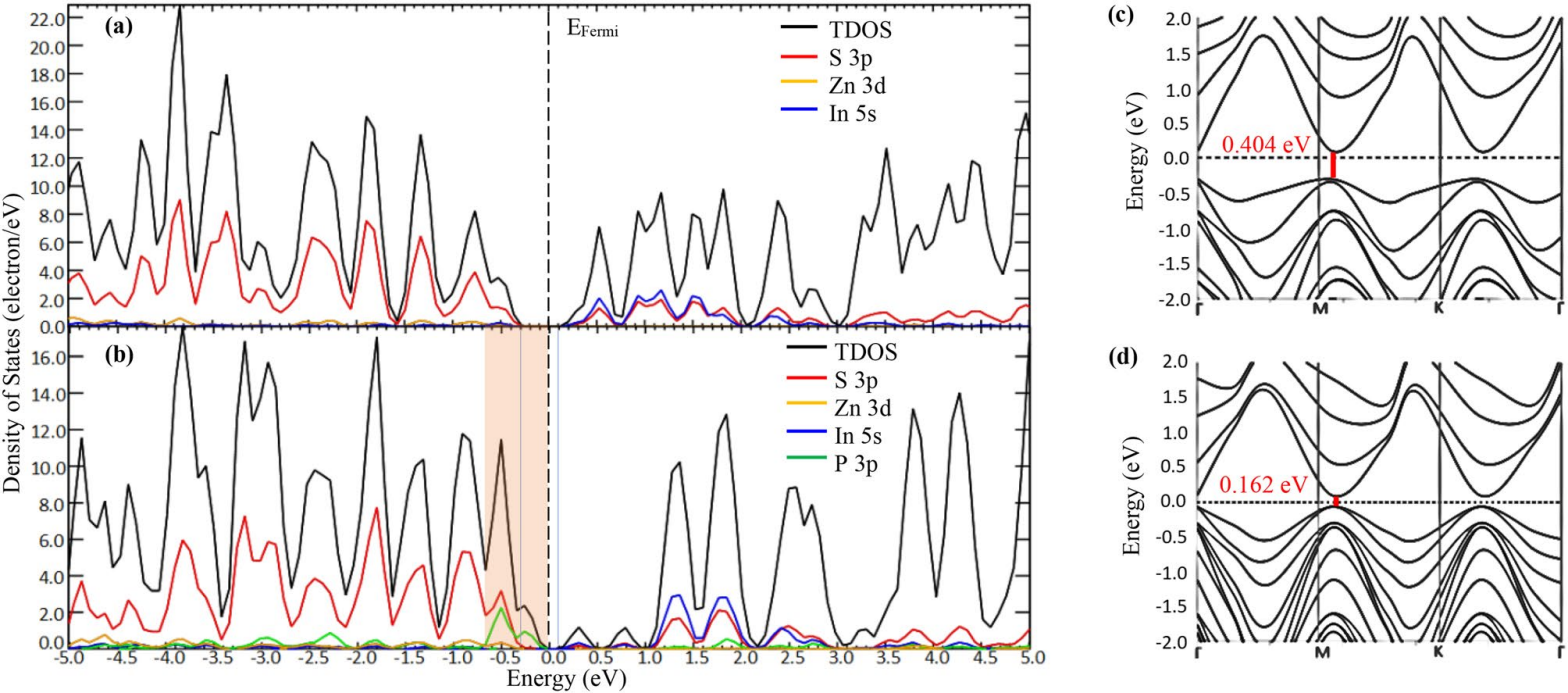
Calculated DOS of (**a**) pristine ZIS, and (**b**) P-ZIS (SPD-3). E_Fermi_ = Fermi level (set to 0 eV). Vertical blue lines represent VBM and CBM of pristine structure respectively for comparison. Light orange shading marks the increased DOS at VBM due to P-doping. Calculated band structures of (**c**) pristine ZIS and (**d**) P-ZIS (SPD-3) near VBM and CBM.

**Figure 4:**
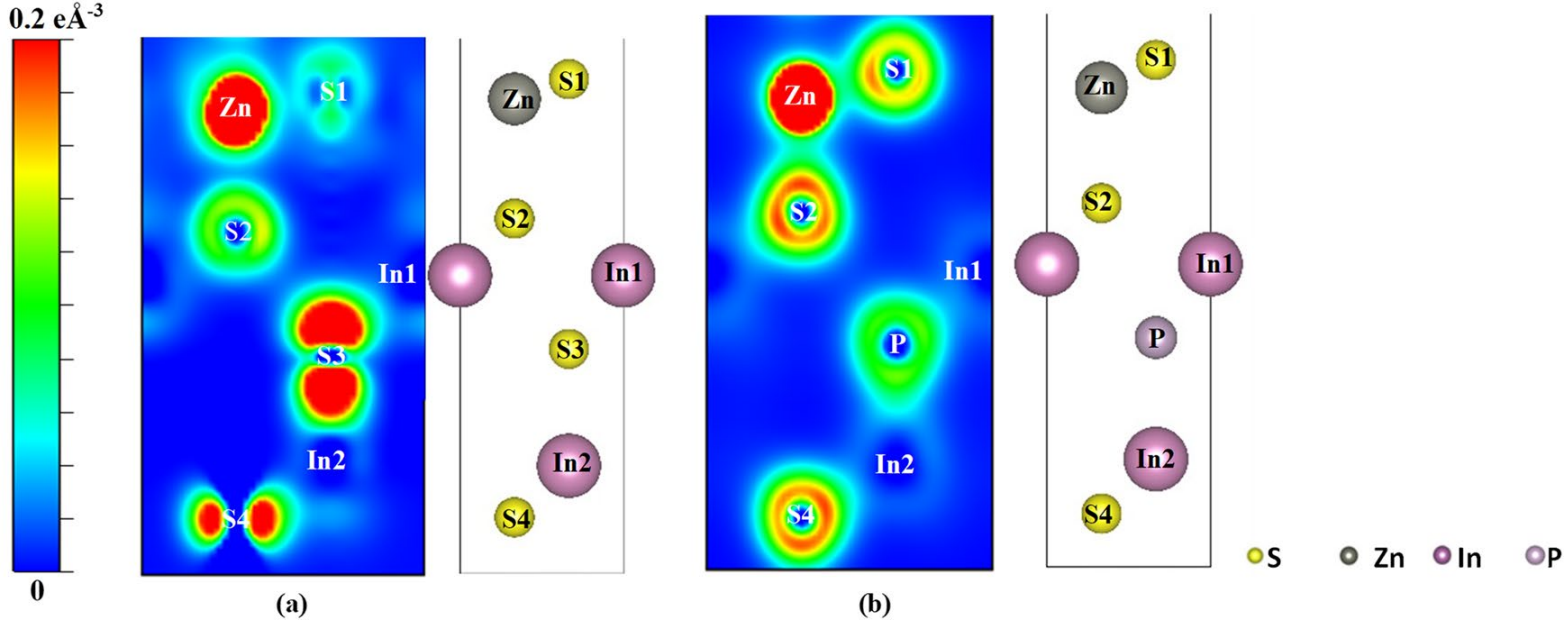
Charge density distribution of (**a**) pristine ZIS and (**b**) P-doped ZIS (SPD-3) at (100) doped monolayer. Respective lattice structure is provided at the side for ease of comparison.

Based on Table 1, the formation energy calculated for each of the eight cases had a negative value, which inferred that all of the IPD and SPD processes were energetically favourable. Possible IPD scenarios were considered at 4 different sites with interstitial spaces larger than the P atom. After lattice relaxation computation, significant distortions were observed across all IPD structures (see Fig. S3). As shown in Table 1, the calculated formation energies for IPD structures (ranging from − 0.991 to − 1.172 eV) were much higher than those of SPD structures, which suggested that IPD generally created less favourable structures. On the other hand, all relaxed SPD structures experienced little to no lattice distortion due to the similar atomic radii (~ 100 nm) between S and P atoms (see Fig. S3). Consequently, the calculated formation energies for SPDs (ranging from − 2.011 to − 2.082 eV) were comparatively lower than IPDs, with SPD-3 exhibiting the lowest value. In short, it could be concluded that the substitutional doping of P-atom (replacing intrinsic S3 atom) on ZIS gave rise to the most stable structure, as evidenced by its lowest formation energy of − 2.082 eV. It was believed that the breaking of intrinsic In-S bonds and the formation of new In-P bonds around the In atoms in ZIS were favoured. Thus, all subsequent analyses and studies were performed on the SPD-3 structure. Figures 2, 3 and 4 show the comparison of lattice structures, DOS and charged density distributions between pristine ZIS and P-ZIS (SPD-3) to unravel the potential effects of P-doping towards photocatalytic water splitting.

Comparing the relaxed structures of ZIS and P-ZIS (SPD-3) in Fig. 2, no significant lattice distortions were found. However, a larger interatomic spacing was observed when P atoms substituted the intrinsic S atoms at the bottom layer. This was attributed to the replacement of smaller S atoms with larger P atoms in the P-ZIS structure. The observation also coincided with the negative shift in (110) and (120) peaks in the powder diffraction pattern of P-ZIS, which indicated an increment in lattice spacing as compared to that of pristine ZIS (see Fig. S2). Contrastingly, the interatomic spacing around S1 and S1’ atoms in P-ZIS were found to be reduced, with a slight positive shift of the (006) facet. Furthermore, the lattice bilayer thickness and interlayer spacing were also reduced to 2.477 nm (− 0.36%) and 0.396 nm (− 1.25%), respectively. Apart from those, all other characteristic peaks of P-ZIS were well-aligned to the pristine ZIS structure. The difference in the relative intensity between both patterns could be ascribed to the additional stress and strain imposed by the doping of P atoms^28^.

The calculated DOS and band structure for both pristine ZIS and P-ZIS (SPD-3) are shown in Fig. 3. The band gap energy of pristine ZIS was found to be approximately 0.404 eV (see Fig. 3a, c). Although hybrid function HSE06 was used for band gap and DOS calculations, the results were clearly underestimated as the experimental band gap energy of ZIS should be roughly 2.40 eV^25^. It is known that using DFT to calculate the band gap of complex structures tends to have lower precision. Similar observations were also reported in other papers, where relatively smaller band gaps for ZIS (ranging from ~ 0 to 0.916 eV) were found using DFT simulation^29,31,48,49^. Moreover, the electronic states near the VBM were mainly composed of Zn-3d and S-3p orbitals, while the electronic states near the CBM were contributed by In-5 s and S-3p orbitals of other S atoms. According to Zhang et al. ^48^, such spatial separation of electronic states near the VBM and CBM could potentially give rise to errors and an underestimation of band gap energies.

Figure 3b shows the calculated DOS of P-ZIS, where a variation of band edge was detected following P-doping. It can be observed that the VBM upshifted by 0.242 eV, following by a significant increase in electron density near the VBM associated to the additional P-3p orbital (see orange shading in Fig. 3b). As the CBM remained unchanged, P-ZIS exhibited an overall reduction in band gap energy to 0.162 eV (− 59.9%). This finding was consistent with other DFT studies conducted over anion-doped (O and N) ZIS, where only an upshift of VBM was observed with no changes to the CBM and without the presence of any midgap state^31,51^. Additionally, P-ZIS exhibited different band structures from pristine ZIS (see Fig. 3c, d) owing to the contribution from the atomic orbitals^28^, which further demonstrates that P-doping could alter the electronic properties of ZIS. In short, the reduction in band gap energy could improve the photo-responsiveness of P-ZIS, by harvesting a broader range of the solar spectrum for photocatalytic applications. Moreover, the increase in electron density near the VBM indicates that more ground-state electrons would be available for photoexcitation and subsequent participation in HER upon light irradiation. Simultaneously, the additional states near the VBM close to the Fermi level could enhance the metallic conductivity of P-ZIS, thereby facilitating the mobility of holes and further inhibiting the recombination of photoexcited charge carriers for augmented performance in HER^51^.

Figure 4 reveals the charge density distribution around the atoms in ZIS and P-ZIS at the doping layer. It is clear that the S3 atom possessed high charge distribution, which was replaced by P atom via substitutional doping. Because the electronic states near the CBM were primarily contributed by the S-3p orbital, such substitution step resulted in the decrease of DOS near the CBM of P-ZIS. Similarly, the introduction of P atom into ZIS contributed to higher charge densities near the VBM in the calculated DOS. It is evident that after P-doping, the charge density distribution around S atoms (potential HER active sites) increased significantly due to the delocalization of charges. This would potentially improve the interaction between hydrogen atoms with potential active sites, which would consequently enhance hydrogen adsorption and the photocatalytic performance towards HER.

### Water interaction study

During photocatalytic water splitting, the adsorption of water molecules onto the photocatalyst surface serves as an important step for HER and OER. This is critical for providing H^+^ atoms for the reductive generation of H_2_ molecules, while simultaneously driving the formation of surface hydroxyl radicals to generate O_2_ molecules. Therefore, the interaction and adsorption behaviour of water molecules on pristine ZIS and P-ZIS structures were analysed via DFT. The bonding strength of water adsorption energy ($\Delta E_{{H_{2} O^{*} }}$) was calculated (see Supplementary Information online for calculation details). By definition, a more negative value of $\Delta E_{{H_{2} O^{*} }}$ would imply a more exothermic adsorption process, and thus, a stronger interaction between the H_2_O molecule and the photocatalyst surface^43^. In addition, the effect of P-doping on H_2_O interaction was also analysed on the basis of the changes in bond angle and bond length of the adsorbed H_2_O molecule.

**Figure 5:**
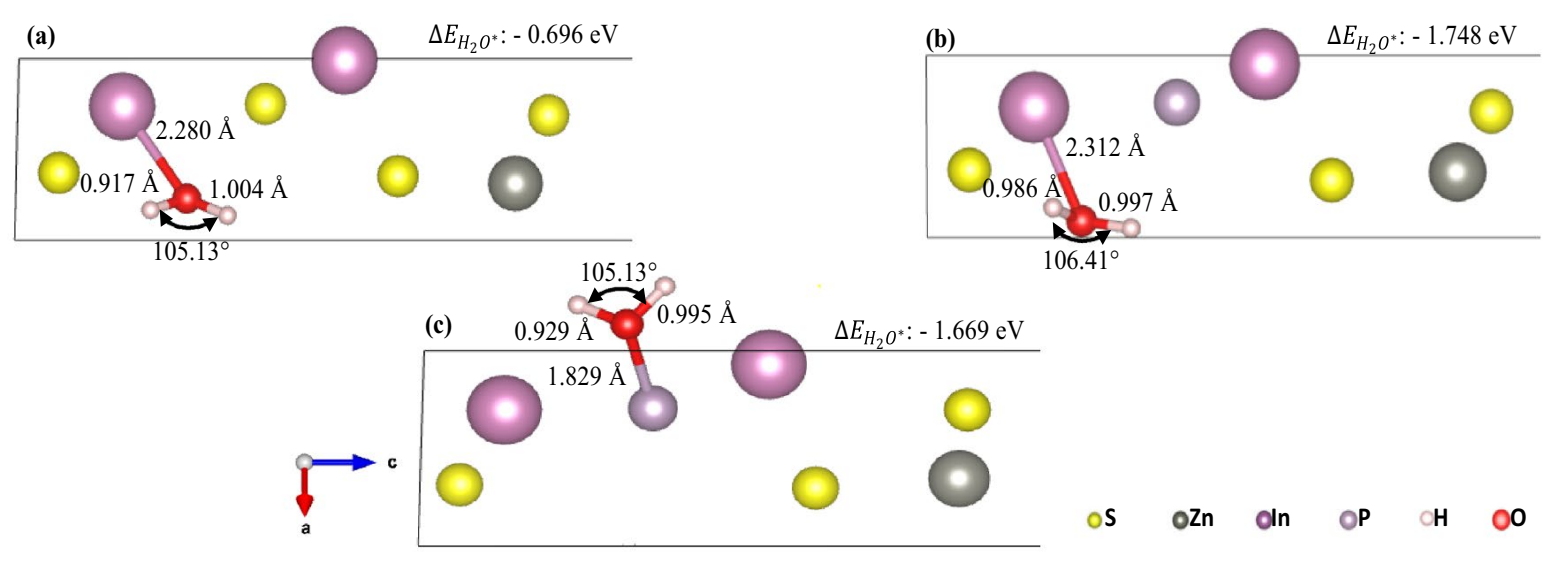
Water molecule interaction with (**a**) In2 from ZIS (110), (**b**) In2 from P-ZIS (110), and (**c**) P from P-ZIS (100). For ease of visualization, only interactive O bonding is shown.

**Figure 6:**
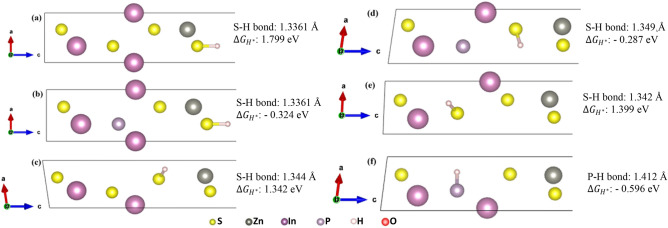
Relaxed monolayer structures, $\Delta G_{{H^{*} }}$ and hydrogen interactive bond length for (**a**) ZIS (H* on S1), (**b**) P-ZIS (H* on S1), (**c**) ZIS (H* on S2), (**d**) P-ZIS (H* on S2), (**e**) ZIS (H* on S3), and (**f**) P-ZIS (H* on P). For ease of visualization, only S–H or P–H bond are shown.

All possible sites (Zn, In, S and P) for H_2_O adsorption in both pristine ZIS and P-ZIS structures were investigated. Based on a study by Li et al.^45^, H_2_O molecules have the tendency to be adsorbed onto positively charged atoms or atoms with lower localized electron density. As all S atoms are non-positively charged, there was no adsorption of H_2_O molecule on the S atoms observed, irrespective of the initial position of the H_2_O molecule. Similarly, no H_2_O molecule was found to be adsorbed onto the Zn atoms on both pristine ZIS and P-ZIS. This could be ascribed to the high localization electron cloud surrounding the Zn atom (see Fig. 5), which induced repulsion with the free H_2_O molecule and prevented its adsorption. These findings indicated that neither S nor Zn atom could serve as active sites for the adsorption of H_2_O. On the other hand, the adsorption and activation of H_2_O molecule were observed on In2 atoms in both pristine ZIS and P-ZIS, as well as P-atoms in P-ZIS (see Fig. 6). This suggested that both In2 and P could serve as activation regions for H_2_O and potentially act as reactive sites for OER. Interestingly, no H_2_O molecule was adsorbed onto In1, which could be due to insufficient interatomic spacing for H_2_O adsorption. It could be observed that following the introduction of H_2_O molecule towards In1 atom, the relaxed ZIS and P-ZIS structures showed great distortion in their crystal lattices (see Fig. S5c, d).

The bond angle and O–H bond length of an isolated free H_2_O molecule are ~ 104.53° and ~ 0.973 Å, respectively^52,53^. As can be seen from Fig. 5a, the bond length of In2-O in pristine ZIS was 2.280 Å with a calculated $\Delta E_{{H_{2} O^{*} }}$ of − 0.696 eV. One of the O–H bonds (1.004 Å) of the adsorbed H_2_O was longer than that in a free H_2_O molecule (0.937 Å), which suggested the activation of H_2_O upon adsorption. This was further supported by the bond angle of adsorbed H_2_O (106.85°), being greater than that of a free H_2_O molecule (104.53°). However, the other O–H bond of the H_2_O molecule was found to have shortened to 0.917 Å, implying that a higher energy would be required for the subsequent step in OER. P-doping was found to have improved the interaction between H_2_O and In2 atom in P-ZIS as the calculated $\Delta E_{{H_{2} O^{*} }}$ value was significantly more negative at − 1.748 eV. Both O–H bonds of the adsorbed H_2_O molecule were also found to be stretched to 0.997 Å and 0.986 Å, with an increase in bond angle to 106.41° (see Fig. 5b). These could further attest the successful activation of H_2_O molecules for OER. It should be noted that even though one of the stretched O–H bonds from P-ZIS interaction (0.997 Å) was slightly shorter than that of ZIS (1.004 Å), the other O–H bond was significantly stretched to 0.986 Å. This would facilitate the OER initialization phase and consequently lead to an overall improvement in OER performance on the In2 active site of P-ZIS. The cleavage of both O–H bonds from the H_2_O molecule could result in the production of intermediate species prior to the formation of O_2_ molecules; while promoting HER through the production of H^+^ atoms.

In addition to In2, P-doping could also provide an additional active site for H_2_O and improve the photocatalytic performance of P-ZIS towards OER. Intrinsically, no H_2_O interaction or adsorption would be observed on the S atom. However, upon substituting S3 with P, the H_2_O molecule was found to have adsorbed onto the P atom, thereby giving rise to a new active site for OER. Moreover, the negative value of $\Delta E_{{H_{2} O^{*} }}$ on P (− 1.669 eV) suggested an energetically favourable reaction. It can be observed from Fig. 5c that the adsorption of H_2_O molecule onto the P site resulted in the stretching of O–H bond to 0.995 Å, with a bond angle expansion of 105.13°. However, the other O–H bond was found to have shortened to 0.929 Å, which suggested that the P atom would serve only as a secondary active site to In2 in the P-ZIS structure.

### Hydrogen evolution reaction and adsorption study

Generally, HER requires the reduction of two H^+^ ions to produce one H_2_ molecule as described in Eq. (1). Considering the thermodynamic process on the photocatalyst surface, HER involves the adsorption and binding of H atom (H*) onto the active site of photocatalyst, followed by re-combinative desorption of molecular H_2_ gas. Hence the differential binding energy of H ($\Delta E_{{H^{*} }}$) and adsorption free energy of H* (∆G_H_*) can be calculated^41,54^ (see Supplementary Information online for calculation details). Close-to-zero value of ∆G_H_* implies that reaction barriers in both adsorption and desorption steps are compromised which favours HER, serving as an indicator for good photocatalyst for HER^43^.

1 $${\text{Hydrogen}}\,{\text{Evolution}}\,{\text{Reaction}}\,{\text{(HER)}}:2{\text{H}}^{ + } + 2e^{ - } \to {\text{H}}_{2}$$

**Figure 7:**
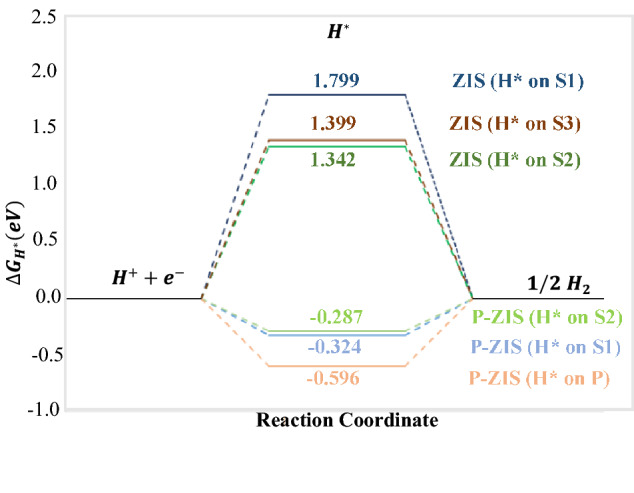
Free energy diagram for hydrogen evolution for each site on ZIS and P-ZIS.

To evaluate the hydrogen adsorption behaviours and potential HER performances, the surface properties of monolayer pristine ZIS and P-ZIS were analysed in detail. The analysis was carried out to elucidate the effect of P-doping on the hydrogen adsorption behaviour over the least favourable site (S1), the most favourable site (S2) as well as the doping site (S3 for ZIS and P for P-ZIS). To facilitate the visualization of hydrogen adsorption, 6 different relaxed structures and their corresponding hydrogen interactive bond lengths obtained from DFT are depicted in Fig. 6. The free energy diagram for HER for each site on ZIS and P-ZIS is displayed in Fig. 7.

Based on the calculation conducted for pristine ZIS (Fig. 7), it can be confirmed that S1 was the least favourable HER active site (high $\Delta G_{{H^{*} }}$ of 1.799 eV) while S2 was the most favourable HER active site (low $\Delta G_{{H^{*} }}$ of 1.342 eV). After performing P-doping, the charge density distributions around the least (S1) and most favourable (S2) active sites were observed to have drastically improved (Fig. 4), which could collectively enhance the interaction and adsorption of H* for HER to take place^55^. From Figs. 6 and 7, the $\Delta G_{{H^{*} }}$ on S1 was found to have reduced from 1.799 (for pristine ZIS) to − 0.324 eV (for P-ZIS). The latter, with a value much closer to zero, indicated that the adsorption–desorption barrier was significantly reduced, which rendered P-ZIS more favourable for H* adsorption. This affirmed that P-doping could result in the improvement of HER performance on the least favourable S1 site. Similarly, the increment of charge density distribution after P-doping on S2 further improved the favourability of H* adsorption onto the most favourable active site (as indicated by the close-to-zero $\Delta G_{{H^{*} }}$). The significant reduction of the adsorption–desorption barrier for P-ZIS was attributed to the elongated S–H interactive bond, which in turn eased the desorption of H* to form H_2_. Likewise, the substitution of P atom favoured the adsorption of H* as indicated by its closer-to-zero $\Delta G_{{H^{*} }}$ of − 0.596 eV as compared to the S3 atom (1.399 eV). The relatively longer P–H bond over S3-H bond could further promote the desorption of H* for H_2_ formation and subsequently improve the HER activity. In short, it is clear that P-doping in ZIS enhances HER performance by (1) reducing H* adsorption–desorption barriers for the generation of H_2_, (2) increasing electron density near HER active sites and (3) inhibiting electron–hole pairs recombination as previously discussed.

### Oxygen evolution reaction and adsorption study

In the overall photocatalytic water splitting process, OER is considerably more complicated than HER as it involves a four-electron transfer pathway. Firstly, H_2_O molecule is adsorbed onto the surface active sites, which is then followed by the formation of 3 different oxygenated reaction intermediates (HO*, O* and HOO*). Subsequently, molecular O_2_ is formed from the HOO* species, in which the reaction occurs at coordinatively unsaturated surface active sites. In short, OER consists of 4 elementary reaction steps (OER1, OER2, OER3 and OER4) as listed in Eqs. (2–5) ^43,56^:

2 $${\text{OER}}\,{1}:\,H_{2} O + * \rightleftharpoons HO^{*} + H^{ + } + e^{ - }$$

3 $${\text{OER}}\,{2}:\,HO^{*} \, \rightleftharpoons O^{*} + H^{ + } + e^{ - }$$

4 $${\text{OER}}\,{3}:\,O^{*} + H_{2} O \rightleftharpoons HOO^{*} + H^{ + } + e^{ - }$$

5 $${\text{OER}}\,{4:}\,HOO^{*} \rightleftharpoons { * } + O_{2} + H^{ + } + e^{ - }$$

where * represents the surface active site, and X* denotes an adsorbed intermediate (X) on the surface active site. The Gibbs free energy for each OER reaction step (∆G_OER_) was evaluated based on the corresponding E_f_ of each component and correction factors based on DFT computation^43,56–58^ (see Supplementary Information online for calculation details). For comparison, the overpotential $\left( {\eta^{OER} } \right)$ required to achieve a downhill trend for all OER free-energy steps at standard equilibrium potential (where external bias U = 1.23 V ^56^) was evaluated using Eq. (6). To be classified as a good OER catalyst, the calculated $\eta^{OER}$ should be as low as possible^43^.

6 $${\text{OER}}\,{\text{Overpotential}}:\eta^{OER} = \frac{{Max\left\{ {\left| {\Delta G_{OER1} \left| , \right|\Delta G_{OER2} \left| , \right|\Delta G_{OER3} \left| , \right|\Delta G_{OER4} } \right|} \right\}}}{e}\left. \right|_{U = 1.23 V}$$

**Figure 8:**
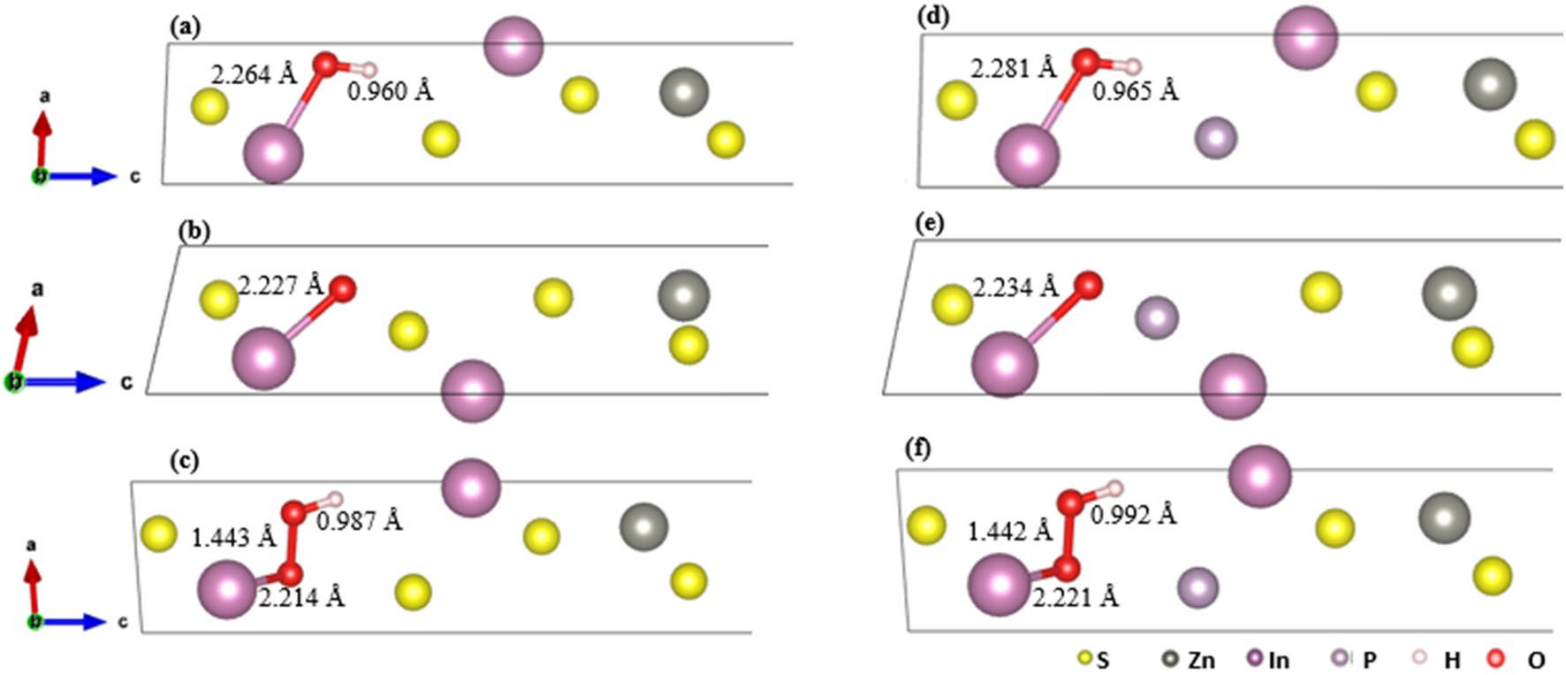
Intermediate reaction steps for monolayer ZIS forming (**a**) HO*, (**b**) O* and (**c**) HOO*, as well as for monolayer P-ZIS forming (**d**) HO*, (**e**) O* and (**f**) HOO*.

**Figure 9:**
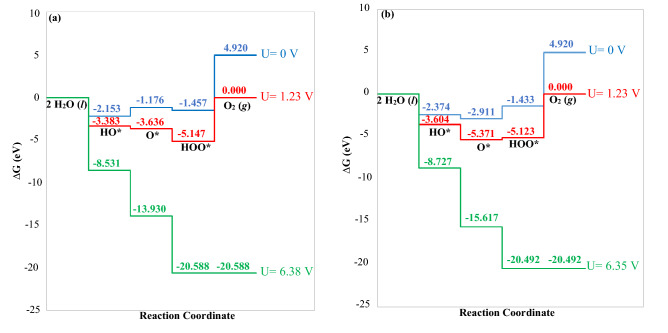
Free energy diagram for oxygen evolution on (**a**) ZIS and (**b**) P-ZIS.

Herein OER analysis was carried out for both pristine ZIS and P-ZIS to study the effect of P-doping on OER performance in In2 active site. The different reaction intermediate steps are shown in Fig. 8. The free energy diagram for OER pathway is also reflected in Fig. 9, with each coordinate Gibb’s free energy of reaction and ∆G_OER_ of corresponding pathway tabulated in Tables S1 and S2 respectively.

Focusing on pristine ZIS, it was previously mentioned that the In2-O bond for adsorbed H_2_O was 2.280 Å (Fig. 5a). When the O–H bond was firstly cleaved via deprotonation of H atom to form HO*, the In2-O bond length was found to decrease to 2.264 Å (Fig. 8a). The further cleavage of the 0.960 Å O–H bond via subsequent H deprotonation to form O* led to an additional reduction in the In2-O bond length to 2.227 Å (Fig. 8b). When O* reacted with free H_2_O molecule to form OOH*, the In2-O bond length decreased to 2.214 Å (Fig. 8c). The OER on pristine ZIS was completed following the cleavage of the 0.987 Å O–H bond via the last H deprotonation step. This was followed by the desorption of O_2_ from the ZIS surface, which resulted in the production of a free O_2_ molecule.

The initial In2-O bond for adsorbed H_2_O on the P-ZIS structure was previously found to be 2.312 Å (see Fig. 5b). After the first cleavage of the O–H bond to form HO*, the In2-O bond length decreased to 2.281 Å as shown in Fig. 8d. For P-ZIS, the subsequent deprotonation of H atom to form O* was deduced to be relatively easier compared to ZIS due to the longer O–H bond of 0.965 Å as shown in Fig. 8e. Next, O* on P-ZIS reacted with the free H_2_O molecule to form OOH* as shown in Fig. 8f. In order to produce free O_2_ molecule, the O–H bond must be cleaved via deprotonation, followed by the desorption of O_2_ from P-ZIS. Similarly, as both of the O–H (0.992 Å) and In2-O (2.221 Å) bond lengths for P-ZIS were comparatively longer than those in ZIS, easier H deprotonation and O_2_ desorption were therefore expected over P-ZIS. These could collectively lead to improved OER performance of P-ZIS as compared to its pristine counterpart.

To further unravel the mechanism of OER, the free energy pathway diagrams for the process over pristine ZIS and P-ZIS were constructed and analysed. For pristine ZIS as presented in Fig. 9a, it can be seen that OER2 and OER4 (as in Eq. (3) and (5) respectively) presented an uphill process with no applied bias (U = 0 V). At the standard equilibrium potential for OER (U = 1.23 V), OER4 remained uphill with a $\eta^{OER}$ value of 5.147 V. This implied that the desorption of O_2_ from the photocatalyst surface acted as the rate determining step (RDS). As shown in Fig. 8, the In2-O bond for HOO* was the shortest among all intermediate steps, which led to its strong adsorption onto In2. To achieve a downhill trend for each step, a bias U of 6.38 V was required. The relatively high $\eta^{OER}$ suggested that OER over pristine ZIS was energetically unfavourable. Figure 9b depicts the free energy diagram for OER over P-ZIS. It can be seen that OER3 and OER4 (as in Eqs. (4) and (5) respectively) presented an uphill process in the absence of an applied bias U. Similar to pristine ZIS, OER4 was observed to be the RDS at the standard equilibrium potential for OER (U = 1.23 V) with a $\eta^{OER}$ value of 5.123 V. It is noteworthy that even though P-doping reduced the overpotential by 0.024 V and out-performed pristine ZIS in the OER intermediate steps, the high $\eta^{OER}$ (> 1 V) indicated that P-ZIS was not energetically efficient in promoting OER for O_2_ production via overall water splitting^56^. Owing to the infeasibility of OER4’s uphill process, the HOO* radical is anticipated to undergo oxidative recombination with H^+^ to produce H_2_O_2_ instead^59,60^. In short, P-ZIS has the potential to efficiently drive solar water splitting to generate H_2_; however, it does not result in the generation of the desired O_2_ product.

## Conclusion

In this work, detailed DFT calculations and computation analysis were successfully performed to fully unravel the nature of P-doping on ZIS. The most stable P-doped ZIS structure was obtained via the replacement of S3 atoms with substitutional doping of P. The calculated E_f_ of − 2.082 eV suggested that the formation of P-ZIS was energetically favourable. A few key points were observed following the doping of P atoms: (1) The band gap of P-ZIS was reduced, indicating enhanced photo-responsiveness; (2) The VBM was upshifted close to the Fermi level due to P-3p orbital contributions, which led to enhanced hole mobility and charge carrier separation; (3) The electron density near the VBM of P-ZIS was increased, which provided more ground-state electrons for photoexcitation and subsequent participation in HER; (4) The H* adsorption was drastically improved due to the increase in charge density distribution around the most favourable HER active site (S2); this resulted in the significant reduction in the H* adsorption–desorption barrier, which could in turn enhance HER performance. The P-ZIS photocatalyst not only displayed improved interaction on the intrinsic OER active site (In2), but also introduced P atoms as new reactive sites for H_2_O adsorption. It should be noted that although P-ZIS exhibited a lower overpotential for OER compared to ZIS, its high $\eta^{OER}$ value of 5.123 V indicated that OER was still energetically unfavourable over P-ZIS. All in all, we strongly believe that this work would provide critical insights into development of high performing P-ZIS-based photocatalyst for enhanced H_2_ generation, which could ultimately bring about the successful commercialization of solar-driven water splitting in the near future.

## Supplementary Information

Supplementary Information.
