# Supplementary material for: Insights from density functional theory calculations on heteroatom P-doped ZnIn2S4 bilayer nanosheets with atomic-level charge steering for photocatalytic water splitting
Source: Sci Rep. 2022 Feb 4;12:1927. doi: 10.1038/s41598-022-05740-8 (PMC8817050; doi:10.1038/s41598-022-05740-8)
Supplement: Supplementary file 1 — Supplementary Information. [file 41598_2022_5740_MOESM1_ESM.docx]

**Supplementary Information**

**Insights from Density Functional Theory calculations on heteroatom P-doped ZnIn_2_S_4_ bilayer nanosheets with atomic-level charge steering for photocatalytic water splitting**

*Wei-Kean Chong, Boon-Junn Ng, Chen-Chen Er, Lling-Lling Tan, Siang-Piao Chai**

Multidisciplinary Platform of Advanced Engineering, Chemical Engineering Discipline, School of Engineering, Monash University, Jalan Lagoon Selatan, Bandar Sunway, 47500 Selangor, Malaysia

* Corresponding author.

Email address: [chai.siang.piao@monash.edu](mailto:chai.siang.piao@monash.edu) (S.-P. Chai)

**Supplementary Information**

**Computational and calculation details**

**Phosphorus doping formation energy calculation:** The formation energy equations for interstitial- and substitutional P-doping are described by **Eq. (S1) – (S2),** where $E_{f}$ represents the formation energy; $E_{P-ZIS}$and $E_{ZIS}$ are the total energy of P-doped and pristine ZIS; $\mu_{P}$ and $\mu_{S}$ are the chemical potential of the P-dopant and substituted S-atom derived from the bulk phase respectively. It should be noted that the lowest formation energy calculated would correspond to the most stable and energetically favourable structure.^1,2^

Interstitial P-doping: $E_{f,IPD}=E_{P-ZIS}-(E_{ZIS}+\mu_{P})$ **(S1)**

Substitutional P-doping: $E_{f,SPD}={(E}_{P-ZIS}+\mu_{S})-(E_{ZIS}+\mu_{P})$ **(S2)**

**Water interaction study:** The bonding strength of water adsorption energy ($\Delta E_{H_{2}O^{*}}$) was calculated using **Eq. (S3)**, where $E_{PC+H_{2}O}$ represents the total energy of a water molecule adsorbed onto the photocatalyst surface, $E_{PC}$ denotes the surface slab energy of the photocatalyst with no water molecule adsorbed, and $E_{H_{2}O}$ is the energy of a free water molecule.

Water adsorption energy: $\Delta E_{H_{2}O^{*}}=E_{PC+H_{2}O}-{(E}_{PC}+E_{H_{2}O})$ **(S3)**

By definition, a more negative value of $\Delta E_{H_{2}O^{*}}$ would imply a more exothermic adsorption process, and thus, a stronger interaction between the H_2_O molecule and the photocatalyst surface^3^.

**Hydrogen evolution reaction and adsorption study: .** Generally, HER requires the reduction of two H^+^ ions in production of one H_2_ molecule as shown in **Eq. (S4)**. Considering the thermodynamic process on the photocatalyst surface, HER involves the adsorption and binding of H atom (H*) onto the active site of photocatalyst, followed by re-combinative desorption of molecular H_2_ gas, hence the differential binding energy of H ($\Delta E_{H^{*}})$can be calculated using **Eq. (S5)**.

Hydrogen Evolution Reaction (HER): $2H^{+}+2e^{-}\to H_{2}$ **(S4)**

Differential binding energy of H: $\Delta E_{H^{*}}=E_{PC+H}-{(E}_{PC}+\frac{1}{2}E_{H_{2}})$ **(S5)**

where $E_{PC+H}, E_{PC}$ and $E_{H_{2}}$ represent the total energy of photocatalyst with one adsorbed H-atom, total energy of photocatalyst without H-atom and energy of gas phase hydrogen molecule, respectively. The adsorption free energy of H* (∆G_H_*), in addition to differential binding energy, can be then calculated using **Eq. (S6)**.

Adsorption free energy of H*: $\Delta G_{H^{*}}=\Delta E_{H^{*}}+\left( \Delta ZPE-T\Delta S \right)_{H^{*}}$ **(S6)**

where $\Delta ZPE$ represents the difference in zero-point energy correction for vibration frequency between absorbed phase (H*) and gas phase (H_2_) and $T\Delta S$ denotes the entropy contribution by the adsorption of ½ H_2_ molecule at T temperature^4^. For metal catalysts, the calculation of ∆G_H_* can be simplified to **Eq. (7)** which is subsequently utilized to describe HER activity and the behaviour of H* on the active
site^4-6^. Close-to-zero value of ∆G_H_* implies that reaction barriers in both adsorption and desorption steps are comprised which favours HER, serving as an indicator for good photocatalyst for HER^3^.

Adsorption free energy of H*: $\Delta G_{H^{*}}=\Delta E_{H^{*}}+0.240 eV$ **(S7)**

**Oxygen evolution reaction and adsorption study.** In the overall photocatalytic water splitting process, OER is considerably more complicated than HER as it involves a four-electron transfer pathway. Firstly, H_2_O molecule is adsorbed onto the surface active sites, which is then followed by the formation of 3 different oxygenated reaction intermediates (HO*, O* and HOO*). Subsequently, molecular O_2_ is formed from the HOO* species, in which the reaction occurs at coordinatively unsaturated surface-active sites. In short, OER consists of 4 elementary reaction steps (OER1, OER2, OER3 and OER4) as listed in **Eq. (S8) – (S11)** ^3,7^:

OER 1: $H_{2}O \left( liq \right)+ * \rightleftharpoons HO^{*}+H^{+}+e^{-}$ **(S8)**

OER 2: $HO^{*}\rightleftharpoons O^{*}+H^{+}+e^{-}$ **(S9)**

OER 3: $O^{*}+H_{2}O\rightleftharpoons HOO^{*}+H^{+}+e^{-}$ **(S10)**

OER 4: $HOO^{*}\rightleftharpoons* +O_{2}+H^{+}+e^{-}$ **(S11)**

where * represents the surface active site, and X* denotes an adsorbed intermediate (X) on the surface active site. To evaluate the Gibbs free energy for each OER reaction step, additional consideration and standardization are accounted. At standard conditions ($T=298 K, P=1 bar$ and $pH=0)$, free energy of H^+^ and e^–^ can be taken as half of the formation energy of H_2._ Besides, theoretical standard Gibbs free energy involving the formation of oxygen will be used as shown in **Eq. (S12)** in order to obtain comparable and consistent results^3,7^.

$\Delta G_{(2H_{2}O\to O_{2}+2H_{2})}=E_{O_{2}}+2E_{H_{2}}-2E_{H_{2}O}+\left( \Delta ZPE+T\Delta S \right)_{\left( 2H_{2}O\to O_{2}+2H_{2} \right)}=4.92 eV$ **(S12)**

After simplification and standardization, the Gibbs free energy (in eV) for each OER step can be calculated as shown in **Eq. (S13) – (S16)**, with correction factors accounted for.

$\Delta G_{OER1}=E_{HO^{*}}+\frac{1}{2}E_{H_{2}}-E_{H_{2}O}-E^{*}+\left( \Delta ZPE-T\Delta S \right)_{OER1}-eU$ **(S13)**

$\Delta G_{OER2}=E_{O^{*}}+\frac{1}{2}E_{H_{2}}-E_{{HO}^{*}}+\left( \Delta ZPE-T\Delta S \right)_{OER2}-eU$ **(S14)**

$\Delta G_{OER3}=E_{HOO^{*}}+\frac{1}{2}E_{H_{2}}-E_{O^{*}}-E_{H_{2}O}+\left( \Delta ZPE-T\Delta S \right)_{OER3}-eU$ **(S15)**

$\Delta G_{OER4}=\left( 4.92+2E_{H_{2}O}-\frac{3}{2}E_{H_{2}} \right)+ E^{*}-E_{HOO^{*}}+\left( \Delta ZPE-T\Delta S \right)_{OER4}-eU$ **(S16)**

in which $E^{*},E_{HO^{*}}, E_{O^{*}}$ and $E_{HOO^{*}}$ denote the calculated energy of clean photocatalyst surface active site and surfaces with adsorbed $HO^{*},O^{*}$ and $HOO^{*}$species, respectively. $E_{H_{2}O},E_{H_{2}}$ and $E_{O_{2}}$ represent the energies of isolated molecules of H_2_O, H_2_ and O_2_, respectively. $\Delta ZPE$ denotes the correction term for the difference in zero-point energy from vibration frequency computation. It is commonly found that ZPE contribution in adsorption is insignificant and has a value lesser than 2 meV ^8^. $T\Delta S$ represents the entropy contribution of each species which can be computed by referring to standard tables for reactants and products in the gas phase^9^, where the entropies for adsorbed atoms or molecules on surface active sites are generally assumed to be zero^3^. $eU$ term is an external bias $U$value accounted on each proton-coupled electron step, whereby U = 0 V for no applied bias and U = 1.23 V during standard equilibrium potential of oxygen revolution^3,7^. For comparison, the overpotential $(\eta^{OER})$ required to achieve all free-energy steps to be downhill for OER at standard equilibrium potential is calculated using **Eq. (S17).** To be classified as a good OER catalyst, calculated $\eta^{OER}$ should be as low as possible^3^.

OER Overpotential: $\eta^{OER}=\frac{Max\left\{ |\Delta G_{OER1}|,|\Delta G_{OER2}|,|\Delta G_{OER3}|,|\Delta G_{OER4}| \right\}}{e}\left. \right|_{U=1.23 V}$ **(S17)**


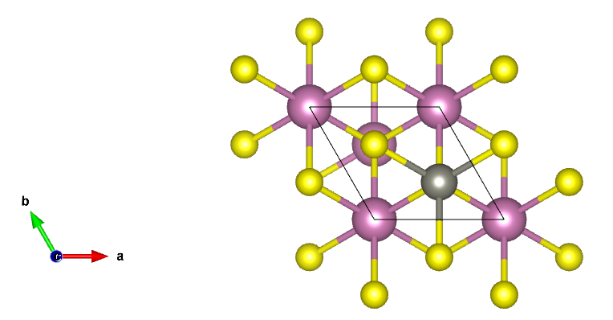

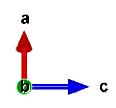

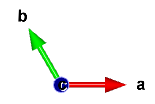


**(c)**

**(d)**


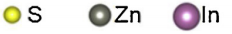

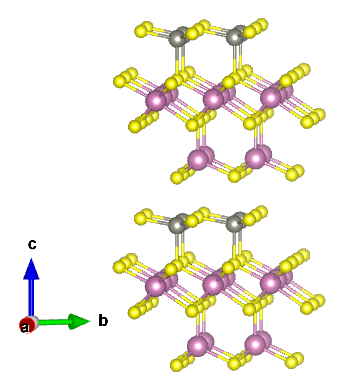


2.550Å

2.300Å

2.544Å

2.417Å

2.496Å

0.401 nm

2.486 nm

(a)


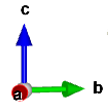


**(b)**


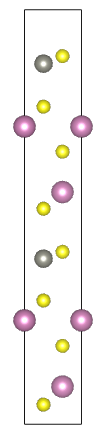


S1’

S1

S2’

S2

S3

S3’

S4

S4’

Zn1

Zn1’

In1

In1’

In2

In2’


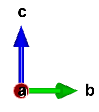

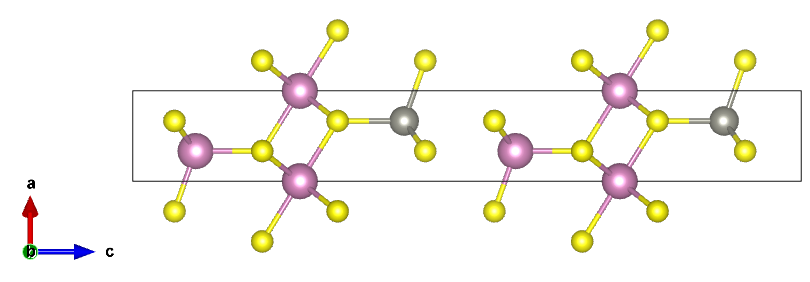


2.481 Å

2.293 Å

2.546 Å

2.399 Å

2.463Å

**Figure S1.** Crystal structure of 2D bilayer ZnIn_2_S_4_ **(a)** with dimensions, and **(b)** excluding bonds for ease of atom visualization. Single ZIS unit cell cut face at **(c)** (110) and **(d)** (001) facet. Labelling of atoms are shown in **(b**) for the ease of discussion in subsequent part.


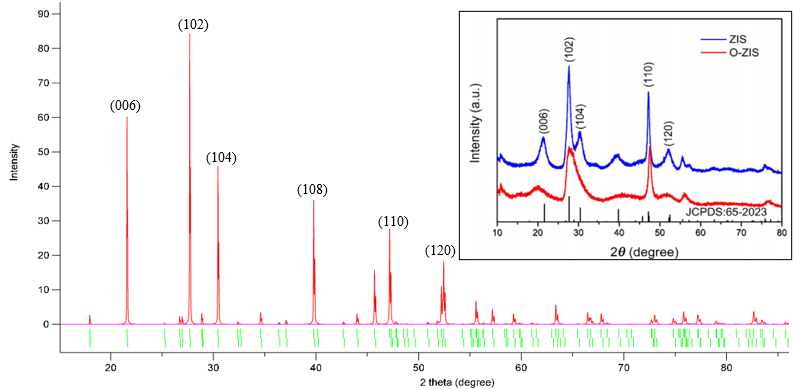


**Figure S2.** Diffraction pattern for simulated ZIS structure and inset displaying X-ray Powder Diffraction (XRD) patterns obtained from literature source by Pan et. al.^10^ (inset blue-lined represents pristine ZIS from the literature source).


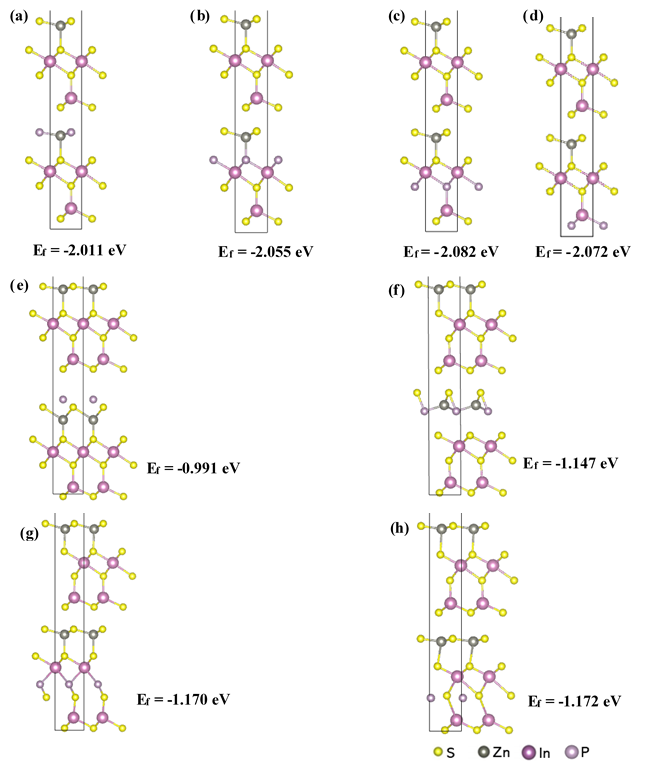


**Figure S3.** Simulated lattice structure of ZIS with single substitutional phosphorus-doped (SPD) models as in **(a)** SPD-1, **(b)** SPD-2, **(c)** SPD-3, and **(d)** SPD-4 as well as single interstitial phosphorus-doped (IPD) models for **(e)** IPD-1, **(f)** IPD-2, **(g)** IPD-3, and **(h)** IPD-4, with its respective formation energy per atom (E_f_).

As can be seen from **Fig. S3(e)**, interstitial doping of P atom into the bilayer spacing greatly increased the space gap to ~0.580 nm (45% increment). The P dopant also altered the Zn1-S1 bonding and created a significant lattice distortion in the bottom layer, which resulted in structural instability as reflected by its highest calculated formation energy of –0.991 eV. Next, the introduction of P atom into IPD site 2 was found to have generated a ‘detached’ structure as depicted in **Fig. S3(f)**, where the intrinsic Zn1-S2 was broken and a new bond between Zn1-P was formed. Such alteration resulted in the formation of a non-stable triplet-layer structure with the second largest formation energy of –1.147 eV. Referring to **Fig. S3(g)**, the extrinsic P atom initially placed in IPD site 3 migrated to the interspacing between In1 and S3 in order to minimize surface energy. The relatively high formation energy of –1.170 eV indicated that the insertion of P atom to form new bonds of Zn1-P and P-S3 within the atomic structure was in fact unstable. **Fig. S3(h)** shows the relaxed IPD structure after introducing P atom into the largest interstitial site (IPD site 4) in the ZIS structure. A distortion of the lattice structure was observed, where Zn1-S2-In1 planar slipped along the negative direction of b-axis while In1-S3-In2 planar slipped towards the positive direction of b-axis. To minimize surface force, the P atom migrated and sat co-planarly with the S3 atom. Even so, the corresponding formation energy was calculated to be –1.172 eV, which was higher than any of the SPD structures, thereby suggesting that IPD-4 was comparatively unstable.


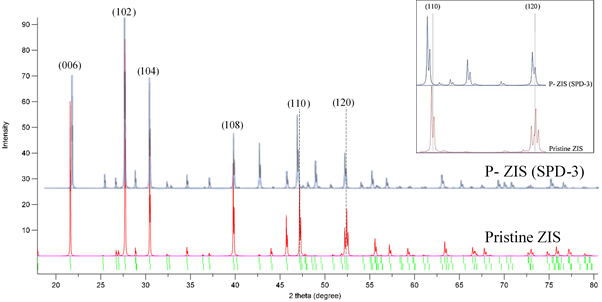


**Figure S4.** Comparison of powder diffraction pattern between simulated pristine ZIS and P-ZIS (SPD-3). Inset shows enlarged (110) and (120) reflection on pristine ZIS and P-ZIS.


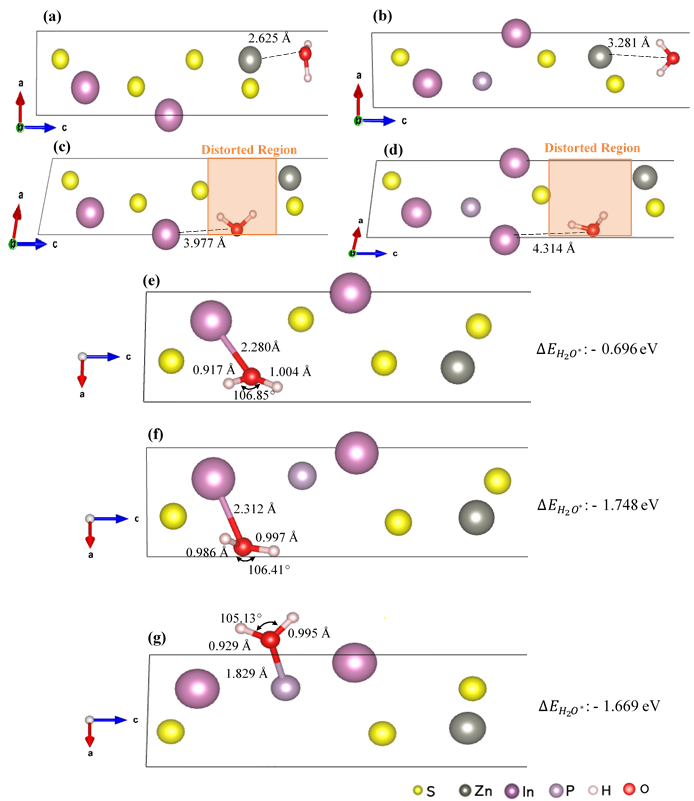


**Figure S5.** Optimized monolayer structure of water molecule interaction with **(a)** Zn1 from ZIS, **(b)** Zn1 from P-ZIS, **(c)** In1 from ZIS, **(d)** In1 from P-ZIS, **(e)** In2 from ZIS, **(f)** In2 from P-ZIS, and **(g)** P from P-ZIS. For ease of visualization, only interactive O bonding is shown. No water interaction is observed in **(a), (b), (c)** and **(d).** $\Delta E_{H_{2}O^{*}}$ is only applicable to adsorbed water molecule onto the structure.

**Table S1.** Reaction Gibb’s Free Energy of each elementary reaction for pristine ZIS and P-ZIS with different external bias U value as well as OER overpotential value.

| Structure | Reaction Gibb’s Free Energy (eV) | | | | External Bias U (V) |
| --- | --- | --- | --- | --- | --- |
|  | $\Delta\boldsymbol{G}_{\boldsymbol{OER}\mathbf{1}}$ | $\Delta\boldsymbol{G}_{\boldsymbol{OER}\mathbf{2}}$ | $\Delta\boldsymbol{G}_{\boldsymbol{OER}\mathbf{3}}$ | $\Delta\boldsymbol{G}_{\boldsymbol{OER}\mathbf{4}}$ |  |
| Pristine ZIS | -2.153 | 0.977 | -0.281 | 6.377 | 0.00 |
|  | -3.383 | -0.253 | -1.511 | 5.147 | 1.23 |
|  | -8.531 | -5.400 | -6.658 | 0.000 | 6.38 |
| P-ZIS  (SPD-3) | -2.374 | -0.537 | 1.478 | 6.353 | 0.00 |
|  | -3.604 | -1.767 | 0.248 | 5.123 | 1.23 |
|  | -8.727 | -6.890 | -4.875 | 0.000 | 6.35 |

**Table S2.** Gibb’s free energy of each reaction coordinate for pristine ZIS and P-ZIS with different external bias U value.

| Structure | Gibb’s Free Energy for Each Reaction Coordinate (eV) | | | | | External Bias U (V) |
| --- | --- | --- | --- | --- | --- | --- |
|  | **H_2_O ^NOTE 1^** | **HO* ^NOTE 2^** | **O* ^NOTE 3^** | **HOO* ^NOTE 4^** | **O_2_ ^NOTE 5^** |  |
| Pristine ZIS | 0.000 | -2.153 | -1.176 | -1.457 | 4.920 | 0.00 |
|  | 0.000 | -3.383 | -3.636 | -5.147 | 0.000 | 1.23 |
|  | 0.000 | -8.531 | -13.930 | -20.588 | -20.588 | 6.38 |
| P-ZIS (SPD-3) | 0.000 | -2.374 | -2.911 | -1.433 | 4.920 | 0.00 |
|  | 0.000 | -3.604 | -5.371 | -5.123 | 0.000 | 1.23 |
|  | 0.000 | -8.727 | -15.617 | -20.492 | -20.492 | 6.35 |
| *NOTE 1: Initialization, Gibbs Free Energy for H_2_O adsorption is taken as 0 eV NOTE 2: Gibbs Free Energy for HO* coordinate = Gibbs Free Energy for H_2_O coordinate + ∆G_OER1_ NOTE 3: Gibbs Free Energy for O* coordinate = Gibbs Free Energy for HO* coordinate + ∆G_OER2_ NOTE 4: Gibbs Free Energy for HOO* coordinate = Gibbs Free Energy for O* coordinate + ∆G_OER3_ NOTE 5: Gibbs Free Energy for O_2_ coordinate = Gibbs Free Energy for HOO* coordinate + ∆G_OER4_* | | | | | | |

# **Reference**

1. Shi, X. *et al.* Inert basal plane activation of two-dimensional ZnIn_2_S_4_ via Ni atom doping for enhanced co-catalyst free photocatalytic hydrogen evolution. *J. Mater. Chem. A* **8**, 13376-13384 (2020).
2. Le, D., Rawal, T. B. & Rahman, T. S. Single-layer MoS_2_ with sulfur vacancies: Structure and catalytic application. *J. Phys. Chem. C* **118**, 5346-5351 (2014).
3. Zhou, X., Dong, H. & Ren, A. M. Exploring the mechanism of water-splitting reaction in NiO_x_/β-Ga_2_O_3_ photocatalysts by first-principles calculations. *Phys. Chem. Chem. Phys.* **18**, 11111-11119 (2016).
4. Shi, X. *et al.* Ultrathin ZnIn_2_S_4_ nanosheets with active (110) facet exposure and efficient charge separation for cocatalyst free photocatalytic hydrogen evolution. *Appl. Catal., B* **265**, 118616 (2020).
5. Yang, W. *et al.* Enhanced photoexcited carrier separation in oxygen-doped ZnIn_2_S_4_ nanosheets for hydrogen evolution. *Angew. Chem. Int. Ed.* **55**, 6716-6720 (2016).
6. Zhu, L., Liu, L., Huang, G. & Zhao, Q. Hydrogen evolution over N-doped CoS_2_ nanosheets enhanced by superaerophobicity and electronic modulation. *Appl. Surf. Sci.* **504**, 144490 (2020).
7. Mukherjee, B. Highly efficient electrocatalyst for oxygen evolution reaction: DFT investigation on transition metal‐tetracyanoquinodimethane monolayer. *ChemistrySelect* **6**, 609-616 (2021).
8. Nguyen-Thuy, T. *et al.* Hydrogen adsorption mechanism of MOF-74 metal–organic frameworks: An insight from first principles calculations. *RSC Adv.* **10**, 43940-43949 (2020).
9. Lide, D. R. *CRC Handbook of Chemistry and Physics*. 84th edn, (CRC Press, 2003).
10. Pan, B. *et al.* Oxygen-doping of ZnIn_2_S_4_ nanosheets towards boosted photocatalytic CO_2_ reduction. *J. Energy Chem.* **57**, 1-9 (2021).

**Figure legends**

1. Figure S1: Crystal structure of 2D bilayer ZnIn_2_S_4_ (a) with dimensions, and (b) excluding bonds for ease of atom visualization. Single ZIS unit cell cut face at (c) (110) and (d) (001) facet. Labelling of atoms are shown in (b) for the ease of discussion in subsequent part.
2. Figure S2: Diffraction pattern for simulated ZIS structure and inset displaying X-ray Powder Diffraction (XRD) patterns obtained from literature source by Pan et. al.^10^ (inset blue-lined represents pristine ZIS from the literature source).
3. Figure S3: Simulated lattice structure of ZIS with single substitutional phosphorus-doped (SPD) models as in **(a)** SPD-1, **(b)** SPD-2, **(c)** SPD-3, and **(d)** SPD-4 as well as single interstitial phosphorus-doped (IPD) models for **(e)** IPD-1, **(f)** IPD-2, **(g)** IPD-3, and **(h)** IPD-4, with its respective formation energy per atom (E_f_).
4. Figure S4: Comparison of powder diffraction pattern between simulated pristine ZIS and P-ZIS (SPD-3). Inset shows enlarged (110) and (120) reflection on pristine ZIS and P-ZIS.
5. Figure S5: Optimized monolayer structure of water molecule interaction with **(a)** Zn1 from ZIS, **(b)** Zn1 from P-ZIS, **(c)** In1 from ZIS, **(d)** In1 from P-ZIS, **(e)** In2 from ZIS, **(f)** In2 from P-ZIS, and **(g)** P from P-ZIS. For ease of visualization, only interactive O bonding is shown. No water interaction is observed in **(a), (b), (c)** and **(d).** $\Delta E_{H_{2}O^{*}}$ is only applicable to adsorbed water molecule onto the structure.

**Table legends**

1. Table S1: Reaction Gibb’s Free Energy of each elementary reaction for pristine ZIS and P-ZIS with different external bias U value as well as OER overpotential value.
2. Table S2: Gibb’s free energy of each reaction coordinate for pristine ZIS and P-ZIS with different external bias U value.
